# A personal perspective on modelling the climate system

**DOI:** 10.1098/rspa.2015.0772

**Published:** 2016-04

**Authors:** T. N. Palmer

**Affiliations:** Department of Physics, University of Oxford, Oxford, UK

**Keywords:** climate prediction, systematic error, seamless prediction, cloud-resolved modelling, International Bureau of Meteorology

## Abstract

Given their increasing relevance for society, I suggest that the climate science community itself does not treat the development of error-free *ab initio* models of the climate system with sufficient urgency. With increasing levels of difficulty, I discuss a number of proposals for speeding up such development. Firstly, I believe that climate science should make better use of the pool of post-PhD talent in mathematics and physics, for developing next-generation climate models. Secondly, I believe there is more scope for the development of modelling systems which link weather and climate prediction more seamlessly. Finally, here in Europe, I call for a new European Programme on Extreme Computing and Climate to advance our ability to simulate climate extremes, and understand the drivers of such extremes. A key goal for such a programme is the development of a 1 km global climate system model to run on the first exascale supercomputers in the early 2020s.

## 1. Introduction

The invitation to contribute to this Special Issue came with the request to write a personal perspective on: ‘How to go forward in solving a problem close to the author’s heart’. I have chosen the problem of how to estimate reliably the impact of anthropogenic carbon emissions on climate. To emphasize that this really is a personal perspective, I use throughout the first person singular, a grammatical form generally avoided in the scientific literature.

A key reason for choosing this topic is that I do not think we take this problem nearly seriously enough. By ‘we’ I do not mean society in general, or indeed the governments that fund research on climate science. Rather, I mean the climate science community. This may seem an extraordinary statement to make given the millions of scientist hours spent developing climate models, running them on large supercomputers, and writing up results in the peer-reviewed literature and for IPCC assessment reports (which themselves consume enormous amounts of time). I therefore need to explain what I mean by this assertion.

Earth’s climate is a nonlinear system *par excellence*. Nowhere is this more manifest than in the dynamics and thermodynamics of the hydrological cycle. Comprehensive climate models represent our best attempt to simulate our changing climate. They do this by attempting to solve *ab initio* the relevant nonlinear laws of physics. These models/simulators play an increasingly important role (i) in providing the science input to global policy on carbon emissions; (ii) in determining the types of infrastructure investments that will have to be made regionally to adapt to climate change; (iii) in assessing whether there is a safe ‘Plan B’ to cool the planet, based, for example, on spraying SO_2_ into the stratosphere (leading to a layer of sulfate aerosol there); (iv) in attributing observed extreme weather events to anthropogenic emissions of greenhouse gases and (v) in extending weather prediction into the seasonal and perhaps even decadal timescales, hence, for example, being able to anticipate specific long-term droughts.

In the view of their relevance in solving urgent real-world problems, it is a matter of concern that climate models continue to exhibit pervasive and systematic errors when compared with observations (see §2). These errors can be as large as the signals predicted to arise from anthropogenic greenhouse gas emissions. Because the climate system is profoundly nonlinear, these errors cannot be simply scaled away, or reliably subtracted out *a posteriori* with some empirical correction based on past observations, when simulating the future climatic response to human carbon emissions.

Of course, climate science is not static, and one expects these model errors to reduce as the models improve (through higher resolution and better sub-grid parametrizations). However, given the urgency of the climate-change issue, a key question is whether this is happening quickly enough. As discussed in §2, despite the vast number of scientist hours spent developing and testing climate models, the improvement in climate models over the last few years has been very modest (indeed, according to some accounts, non-existent). As a result, I myself am convinced that we need urgently to find ways of accelerating developments in the science of climate modelling.

In §§3–5, I will suggest three courses of action that we, the science community, need to take. In §3, I draw attention to a largely untapped pool of mathematical talent that should be recruited into climate science. Modest investment by Research Councils could be transformative in realizing this talent. In §4, I will make the case for a much stronger synergy between weather and climate prediction. It may come as a surprise to an outsider that a strong synergy does not already exist. However, for a number of reasons, not necessarily scientific, the synergy is often not nearly as strong as it should be. Finally, in §5, I want to discuss the establishment of an international meteorological prediction institute; a concept which, it may astonish many readers to learn, Albert Einstein himself advocated back in the 1920s.

Much of the discussion in this paper is predicated on the notion that to first order we do know the equations we wish to solve—it is just that we do not yet have the resources to solve these equations with the accuracy that we would like, and from which society can benefit. Some may argue that this is very much a physicist’s perspective, and that the relevant equations for evolution of biological processes are far from known. I would actually agree with this. For this reason, my focus in this paper is on the importance of simulation and prediction on multi-decadal, rather than multi-century or longer timescales (where biological processes do dominate). Of course, this is not to imply that biological processes are unimportant on these shorter timescales. However, on these shorter timescales they are generally of secondary importance to those associated with the physical hydrological cycle.

## 2. The climatic Turing test

Turing [1] famously asked the question: How could one tell the difference between a human and an artificial intelligence? His answer was (deceptively) simple: ask them questions. If you cannot tell which is which from their answers, then for all practical purposes there is no difference. Very high-resolution limited-area weather forecast models, integrated a day or so from their initial conditions, pass the Turing test on scales larger than the model grid-scale (*ca* 1 km): for example, it is often impossible to tell which is a forecast rainfall field and which is the observed field, as determined from radar echoes. By contrast, it is relatively easy to tell the difference between output from a climate model and the real world at scales larger than the grid-scale of the climate model. On scales close to the grid-scale, even an amateur would notice that cloud structures looked unrealistic. However, more seriously, there are also substantial errors on scales much larger than the grid-scale [2].

**Figure 1.**
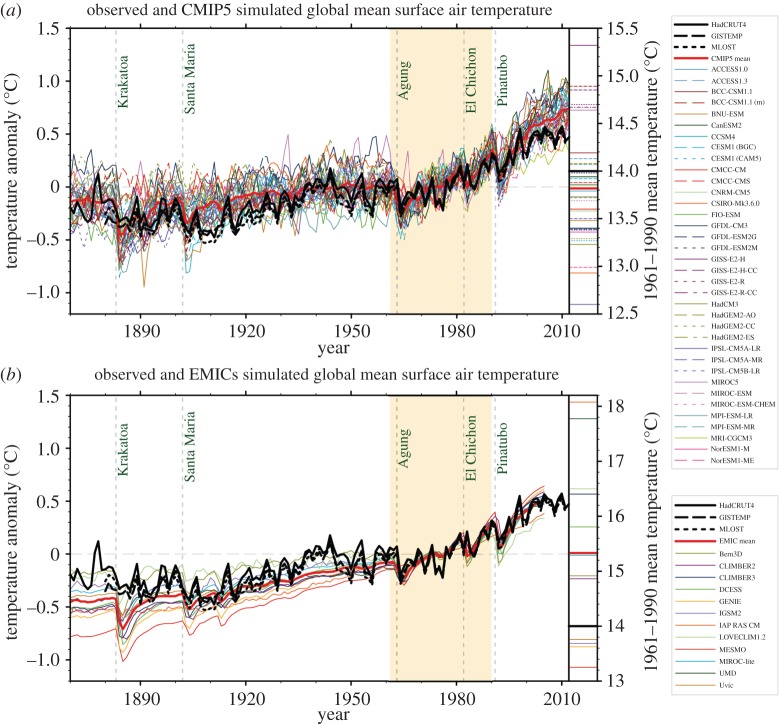
Observed and simulated time series of the anomalies in annual and global mean surface temperature. All anomalies are differences from the 1961 to 1990 time-mean of each individual time series. The reference period 1961–1990 is indicated by yellow shading; vertical dashed grey lines represent times of major volcanic eruptions. (*a*) Single simulations for CMIP5 models (thin lines); multi-model mean (thick red line); different observations (thick black lines). Inset: the global mean surface temperature for the reference period 1961–1990, for each individual model (colours), the CMIP5 multi-model mean (thick red), and the observations. (*b*) Single simulations from available EMIC simulations (thin lines). Observational data are the same as in (*a*). All EMIC simulations ended in 2005 and use the CMIP5 historical forcing scenario. Inset: Same as in (*a*) but for the EMICs (adapted from Flato *et al*. [3]).

As a symptom of this problem, figure 1*a* shows time series from the CMIP5 [4] ensemble of simulations of twentieth century global mean temperature anomaly, together with the corresponding observed anomaly as shown in the IPCC Fifth Assessment Report [3]. The individual model simulations show some scatter among themselves, consistent with the fact that no two models in the CMIP5 multi-model ensemble are identical, and that some variation can be expected in any case by virtue of the chaotic nature of the climate system. By and large, the observations lie within the ensemble of simulations—though the observations lie near the bottom of the ensemble towards the end of the period, consistent with the CMIP5 models having difficulty simulating the strong tropical trade-wind regime associated with the so-called global warming ‘hiatus’. (Figure 1*b* shows a similar set of time series, but for Earth-System models of intermediate complexity. These latter models are not relevant to the discussion in this paper except to note that models of intermediate complexity are not a substitute for the *ab initio* models, though they can help complement such models.)

What may not be obvious from a casual look at figure 1*a*, is that the global mean temperature anomaly for a particular model and a particular year, is the difference between simulated temperature for that year and the 1961–1990 time-mean global mean temperature *for that particular model*. What this means in practice, is that the time-mean systematic error for each model has been subtracted *a posteriori* in the time series shown in figure 1*a*. How large are these systematic errors compared with the observed *ca* 0.75°C trend in twentieth century global mean temperature? This can be gauged by studying the inset on the right-hand side of figure 1*a*. It shows the actual 1961–1990 mean temperature for all the individual models in °C. It can be seen that the range of estimates of 1961–1990 temperature is well over 2°C.

Of course, it is far from easy to simulate global mean temperature from first principles, depending as it does on cloud cover, and hence the hydrological cycle. (It is of course possible to tune a model to have the right surface temperature. Whether the fact that this has not happened is due to scrupulous honesty on the modellers’ part, or whether the models have been tuned to get some other aspect of the simulated climate correct, such as radiative balance, is a moot point.) In any case, the difficulty in simulating the hydrological cycle accurately becomes yet more apparent when comparing a simulated field like rainfall with observations. As noted above, CMIP5 models show substantial large-scale systematic errors in the tropics associated with an excessive equatorial Pacific cold tongue and the so-called ‘double Intertropical Convergence Zone’ [2].

One could perhaps take the view that we should focus attention on those models whose global mean systematic temperature error is relatively small, and discount all others. However, all climate modellers know that to do this would be naive: the models with small systematic error in surface temperature have significant errors in other global fields at surface or higher levels in the atmosphere, implying that the occurrence of small surface temperature error can, and generally does, arise from a partial compensation of errors in the representation of the many physical processes operating in the climate system. This problem of compensation of errors is one of the factors that make climate model development so difficult, and I will return to it in §4.

These systematic errors do not invalidate the use of such climate models in providing scientific input into mitigation policy. These models, our best attempts to solve the laws of physics applied to climate, are quite unequivocal in showing that there is a substantial risk of dangerous, even calamitous, climatic impacts arising from increased levels of atmospheric CO_2_. It is a statement of scientific fact that to reduce this risk will require a reduction of our carbon emissions.

As is well known, the risk of dangerous climate change arises from feedbacks associated primarily with the hydrological cycle as CO_2_ levels increase. However, the magnitude (and for some processes even the sign) of these feedbacks remains profoundly uncertain [5]. If cloud feedbacks in particular turn out to be strongly positive, then climate change could pose an essentially existential threat to large sections of society, unless they can migrate to cooler parts of the planet. (Given the potential for severe conflict arising from mass migration, this option also poses life-threatening risks.) By contrast, if cloud feedbacks turn out to be largely negative—for example, associated with mesoscale organization of convective cloud systems in a warming climate [6]—then the urgency to decarbonize economies by mid-century is reduced, particularly in the developing world, and perhaps more resources can be given to the question of how, with population levels increasing around the world, communities can become more resilient to weather extremes (both natural and human induced).

Given calamitous scenarios at one end of the spectrum of possibilities, and more benign scenarios at the other end, surely climate scientists should be doing everything that science and technology allows to try to reduce uncertainty about future climate. This uncertainty cannot be reduced to zero: there will be uncertainty due to natural variability and scenario uncertainty, and it is completely unrealistic to imagine that model uncertainty will ever be reduced to zero. Nevertheless, to date, development of comprehensive *ab initio* climate models have not helped reduce uncertainties in global warming—the AR5 range is little different to that from estimates made in the 1970s with much simpler models. It seems unlikely that we will be able reduce uncertainty in projections of global warming while model systematic errors are as large as the signals we wish to simulate. Put another way, it seems to me that a necessary condition for substantially reducing uncertainty about future climate is to develop a climate model which passes the climatic Turing test, at least on timescales of a few decades from some observed starting condition.

I think the climate community worldwide does not give enough priority to developing a model which passes the climatic Turing test. We make do with our imperfect models, typically subtracting out the systematic errors against observations when estimating the impact of climate change. Journal referees recognize that this is the best we can do given the current generation of models, and so scientific careers can flourish without having to address the more fundamental question: Why is it so easy to tell the difference between model output and the real world?

Some might disagree and argue instead that our models will improve steadily to the desired level by continuing with the *status quo*where models are developed on an institutional basis, with incremental levels of funding. This might well be so, but what if these steady improvements occur on timescales so slow as to be irrelevant to the needs of the current and next generation? Will climate modelling science have failed in its duty to society?

As evidence that current progress is too slow, consider the improvement in climate models between CMIP3 (feeding into IPCC AR4) and CMIP5 (feeding into IPCC AR5). According to the recent paper by Rauser *et al.* [7]: ‘CMIP5 is not qualitatively better in its ability to represent twentieth century mean state climatology than CMIP3, in the sense that the location and structure of the bias is not fundamentally different, even though the absolute size of the bias is incrementally improved’. The authors conclude that [7], p. 912: ‘CMIP3 and CMIP5 are qualitatively too similar in mean state and response to warrant an automatic generational separation’ and recommend that future multi-model ensemble-based studies combine together CMIP3 and CMIP5 ensembles into one ‘super ensemble’.

In a more recent paper studying tropical biases, Zhang *et al.* [8], see Abstract conclude:

It is found that there is virtually no improvement in all these measures [of tropical circulation] from the CMIP3 ensemble to the CMIP5 ensemble models. …. No progress can be identified in the sub-ensembles of five best models from CMIP3 to CMIP5 even though more models participated in CMIP5; the systematic errors of excessive precipitation and overestimated SST in southeastern Pacific are even worse in the CMIP5 models.

Some might argue that it is a positive thing that the systematic errors of CMIP5 models have at least not increased, because such models generally include more Earth-System processes than corresponding CMIP3 models. However, the sorts of errors referred to here occur on fast timescales, faster than the timescales of the new Earth-Processes.

These matters become even more important if we focus on the regional climate response to increased atmospheric concentrations of greenhouse gases. A crucial aspect of such response is in climate extremes: for example, persistent circulation anomalies which can bring drought and extreme heat for some regions and seasons, and extreme flooding for other regions and seasons. This makes it clear that estimates of future climate change require us to understand the impact of atmospheric CO_2_ on the dynamics of the climate system, and not just its thermodynamics [9,10]. Systematic errors in the strength of troposphere/stratosphere gradients degrade our ability to represent persistent anomalies in jet streams, and systematic errors in tropical circulation fields degrades the teleconnections between the tropics and the extratropics. This makes it extremely difficult to diagnose accurately the real climate drivers of extreme climate anomalies.

It must be emphasized here that I am not in any way criticizing the scientists who do spend much of their time developing climate models. However, considering the number of scientist hours spend developing and evaluating the CMIP5 generation of models, either we do not have sufficient resources to make progress, or we do not make best use of the resources we do have. Personally, I feel the answer is actually both!

Climate science has to do better. How? The next three sections discuss, with increasing levels of difficulty, some strategies.

## 3. Climatologists to physicists: your planet needs you

The most important resource needed to move climate science forward in the twenty-first century is human talent. Developing reliable climate models is not a matter of following some recipe book, it is about finding efficient ways of approximating an (effectively) infinite-dimensional nonlinear system to give approximate but reliable estimates of the future. This process requires imaginative and highly numerate scientists. The urgent need for such talent was brought to light in a recent News article in Nature, entitled ‘Climatologists to physicists: your planet needs you’ [11]. The article raised the question: How to spark enthusiasm for the field of climate science in budding researchers who might otherwise choose, say, astrophysics or cosmology for their focus of research. The article finished with a quote from a fluid-dynamics researcher from the University of Cambridge: ‘Most physics students would rather study with someone like Stephen Hawking, who is a member of our faculty’.

This topic resonates with me because my own PhD was in general relativity theory. My desire to do a PhD in this field was already formed at school, and by the time I was an undergraduate I was completely obsessed with the subject. I cannot imagine anyone being able to deflect me into the field of climate science at that stage of my career. And yet 3 years after my undergraduate degree I was so deflected.

What changed? In part, there was a nagging feeling, which increased with time, that I was not helping society in the slightest by spending large fractions of each day of the week working on abstruse problems that were not only irrelevant to society, but largely unexplainable to the person on the street. Also, I had a growing feeling that the major challenge facing my subject, formulating a theory of quantum gravity, was not going to be solved in the near future (and especially by me). And there was a realization that the mathematics and physics I was learning was relevant in other fields. For example, as part of my PhD work I had begun to study the Principle of Maximum Entropy Production as a technique to understand Hawking Evaporation: the quantum process whereby black holes radiate energy to the outside world. The epiphany which led me into the field of climate science was when geophysicist Raymond Hide, whom I had met by chance during the last year of my PhD, told me of recent work by climatologist Paltridge [12] showing that the properties of Earth’s climate could be derived using the Principle of Maximum Entropy Production!

I was lucky that at the time the Meteorological Office had a policy of taking on new graduates without any requirement for a background in weather or climate science. After a period at the Meteorological Office and a much longer period at the European Centre for medium-Range Weather Forecasts (ECMWF), I am now back in academia. And back in academia, I have begun to realize that there is a considerable pool of PhDs and postdocs, not only in astrophysics and cosmology, but also in high-energy physics, number theory, quantum foundations and other ‘abstruse’ areas of science, who, having achieved their childhood ambitions of doing research in fundamental physics and mathematics, are keen to turn their skills to climate science. They want to save the planet and ask me: How can I make the change to your field?

There is no easy answer to this question. Many of the job openings at National Meteorological Services are for people with experience in specific areas of weather or climate science. Similarly, academic postdoc positions in climate science will require some ‘essential qualifications’ which a number theorist or cosmologist would not have.

So, while agreeing wholeheartedly that climate science needs more mathematicians and physicists, I do not think it is realistic to imagine we can somehow persuade our brightest young scientists not to pursue their dream of quantizing gravity or proving (or disproving) the Riemann Hypothesis, just for the sake of the planet. Indeed I would not even want to try to dissuade them. Like finding the Higgs boson or gravitational waves, I believe strongly that these are important goals for humankind—though less urgent than understanding climate change.

Instead, I believe climate science should be targeting this substantial pool of post-PhD talent. In my experience, such talented scientists are as likely to be challenged by some of the formidable mathematical software engineering problems facing the development of ultra-high resolution climate models, as they might be in doing diagnostic analysis on climate data. That is to say, suitably trained, such talent may well be able to fill some of the current shortages that many climate centres have in software engineering. However, in order for these people to be competitive in the climate science job market, they need to spend a year or two learning the basics of climate and computer science. Because they are bright (you have to be very bright indeed to complete a PhD in number theory), they will pick up these basics extremely quickly and will be able to return investment in such retraining in no time at all.

In these days, where we are all living longer, and where the rules about retirement are being torn up, it makes no sense to take the view that we are largely typecast for the rest of our research lives by the research we do for our PhDs. So, in concluding this section, I call on the National Meteorological Services, the Research Councils (national and international) and the National Academies to jointly fund such retraining schemes—open to PhDs in the fields of physics and mathematics, who wish to refocus their efforts to improve our understanding of the climate system. The cost/benefit ratio is surely minimally small.

## 4. The role of national weather services

In parallel with activities on multi-decadal and centennial climate prediction, the National Meteorological Services (and international institutes such as the ECMWF cf. §5) provide society with forecast products on much shorter timescales—typically from hours to a season or two ahead. What distinguishes such ‘weather forecasts’ from the longer timescale ‘climate projections’? Weather forecasts are essentially initial-value problems: given a sufficiently accurate estimate of the state of the (ocean/atmosphere/land surface) climate system at some initial time *t*_0_, what is the state at some future time *t*_1_. By contrast, climate projections do not depend strongly on the initial state: here one attempts to estimate how the statistics of weather—essentially the geometry of the climate attractor in state space—are affected by some assumed level of carbon emissions into the atmosphere.

However, probe below the surface and it can be seen that thinking about weather and climate forecasting as essential separate activities is not as scientifically meaningful as it might seem. Indeed, I would claim that trying to think about them as separate activities is actually hindering the development of climate science. Not least, weather prediction provides clear-cut metrics of model performance and it should therefore be used in the plethora of techniques needed to help develop a model which can pass the climate Turing test. Below I give two illustrations of this.

### (a) Data assimilation

In order to make accurate estimates of the initial state of the climate system, one clearly needs good quality observations. However, observations are neither of perfect quality, nor, at any particular time, do they cover all parts of the globe. For example, the ability to infer vertical temperature profiles in the atmosphere from satellite-sensed outgoing infrared radiances is strongly compromised by cloud cover. Hence, the best estimates of some initial state combine information from contemporaneous observations with those from earlier times, propagated forward using the equations of motion, i.e. using the weather forecast model. The general framework which allows this is known as data assimilation [13].

The contemporaneous observations will lead to a change in the estimated state of the climate system over that based on older observations and propagated forward in time by the model. This change is referred to as the ‘analysis increment’. One hopes that these analysis increments are not biased in any particular way. However, if the model’s representation of physical processes is erroneous, then the analysis increments will be biased relative to observations. Over the relatively short timescales over which observational information is propagated forward relative to observations in a data assimilation system (typically 6 h), biases in one locality will not have had significant time to propagate to a different locality. Hence studying model errors using a data assimilation system provides a powerful way to isolate errors to specific physical processes. In particular then, by focusing on the development of model error over short timescales, one can minimize the problem of compensation of errors, which, as discussed above, bedevils the diagnosis of model output on longer climate timescales. This technique was introduced by Klinker & Sardeshmukh [14] and used to isolate errors in a parametrization of orographic gravity wave drag. Since then this technique has been an important part of the work at some operational centres, when developing parametrizations [15].

Of course, there are many processes in the climate system that will act on timescales that are much too slow to be diagnosable in data assimilation mode. However, as discussed above, the crucial feedbacks that will determine whether anthropogenic climate change is going to be calamitous or not are associated with clouds, and cloud processes captured by the analysis increment diagnostic. As an example, Rodwell & Palmer [16] showed that certain convective cloud parametrizations whose use in climate models gave rise to particularly large climate sensitivities could be ruled out, because the fit of the analysis increments to contemporaneous observations was particularly poor in regions of strong convection.

Properly used, data assimilation can be a vital tool for understanding the sources of model error, and hence for improving models. It therefore is essential, in my view, for climate institutes to have an integrated data assimilation system in order to be able to make use of this technique for improving models—and for improving representations of the hydrological cycle in particular. However, this is not straightforward: given the many different sources of observational data, both *in situ*and remotely sensed, data assimilation code is immensely complex and running the code occupies a significant fraction of the computer time needed to make a weather forecast.

In practice, this means that climate institutes should be working closely with weather prediction centres, so that the models are closely linked to one another. In this way, information about biases in analysis increments can be used to inform developments in climate model development—especially when the latter can be performed at higher resolution than is possible now. This process of unifying weather and climate prediction systems is generally referred to as ‘seamless’ or unified prediction [17]. In practice, while some seams are inevitable, attempts to minimize the number and extent of seams should be considered a joint goal of weather and climate prediction centres in the future.

### (b) Seasonal forecasting

Seasonal forecasting is an example of an initial-value problem. Unlike weather forecasting a day or two ahead, the oceans and land surface provide much of the crucial information in the initial conditions. While the large-scale systematic errors in weather forecast models play a secondary role in determining the reliability of forecasts a day or two ahead, they play a central role on seasonal (and longer) timescales.

The word ‘reliability’ needs to be unpacked. These days, weather forecasts are based on ensembles of integrations, made by varying the initial conditions and model equations consistent with uncertainty in these elements [18]. An ensemble forecast provides probabilistic predictions of weather events which either occur or do not occur in reality. For example, in the coming season, the seasonal-mean temperature for London will either be above or below average (relative to some suitably defined average). A seasonal forecast may predict that the probability of above-average temperature for London is 65%. What does it mean to say that this is a reliable prediction? If we take a subsample of all the seasonal forecasts where the probability of above-average temperatures lies say in the range 60–70%, then we would expect the observed frequency of occurrence of above-average temperature for this subsample of forecasts to also lie in the range 60–70%. Generalizing this condition to apply to all probability ranges defines a reliable forecast system. Note that reliability is not the same as skill. An unskilful but reliable ensemble forecast should always predict climatological probabilities.

Because models have sizeable systematic errors on seasonal timescales, seasonal forecasts can themselves be unreliable [19]. For example, warmer than average summer temperatures, and colder than average winter temperatures in London are often associated with the occurrence of long-lived blocking anticyclones. However, the climatology of contemporary climate models often shows a deficiency in the simulated frequency of blocking anticyclones over Europe [20]. Such models cannot be expected to produce reliable seasonal forecasts of cold winters or hot summers over Europe.

In an unreliable forecast system, the forecast probability of an event will not be well calibrated against the observed frequency of the event. For example, a model may be overconfident in the likelihood of warm winter temperatures simply, because it is unable to simulate long-lived blocks. An empirical calibration of the forecast probabilities can partially take account of these model errors and make the forecast probabilities somewhat more reliable. However, no quasi-linear empirical calibration scheme can do an entirely satisfactory job, simply because one is dealing with a highly nonlinear system.

An ability to estimate regional climate change will also be impacted if the climate model cannot simulate such blocking events well. This is relevant for a number of climate-related studies. For example, if the number of long-lived blocking anticyclones were to increase as CO_2_ concentrations rise, then the number of winter days when demand for electricity is high, yet the wind is not blowing (nor the sun shining due to the pervasive low-level stratus that often accompanies winter blocks), then the chances of (non-nuclear) renewables alone providing sufficient power supply will be called into question. Also, if the number of blocking anticyclones increases, then it may be necessary to increase reservoir capacity as demand for water will not be easily met by rainfall from cyclonic weather systems. Finally, since droughts and heat waves tend to be associated with long-lived summertime blocks, a model which cannot simulate long-lived blocking well cannot be used to determine the extent to which a particular long-lived drought/heat-wave event can be attributed to anthropogenic climate change.

As such, in a seamless forecast system, where essentially the same model is used in seasonal and longer timescale climate prediction, a measure of the unreliability of seasonal forecasts provides useful (yet incomplete) information to assess the reliability of longer timescale climate forecasts. From the perspective of a climate-change institute, this seasonal forecast information can be obtained ‘cost free’. In particular, a necessary (but not sufficient) condition that longer timescale probabilistic climate forecasts are reliable is that probabilistic seasonal forecasts made with the same model should be reliable. As such, the empirical correction used to make a probabilistic seasonal forecast system more reliable, can also be used to make regional probabilistic climate-change projections more reliable, particularly for quantities like precipitation [21,22].

However, the argument for joining together seasonal and long-term climate forecasting is more than it helps in assessing the reliability of climate projections. The primary bottleneck progressing climate science is lack of (human and computational) resources needed to improve our models. There is so much synergy between the seasonal and longer climate timescale in terms of basic circulation dynamics that the more scientists from these two areas can work closely together, the more the effective resource brought to the table. Research that reduces climate systematic errors on the seasonal timescale—for example, through the use of high-resolution ocean models—is of immediate relevance to the longer climate timescale.

I want to conclude this section with a very specific recommendation. One of the leading multi-model seasonal forecast systems in the world is the EUROSIP system comprising models from ECMWF, the Met Office, Météo-France and the US National Weather Service (information available from the ECMWF web site). This multi-model forecast system arose out of an EU Framework Programme research project (DEMETER) on seasonal climate prediction. The DEMETER project [23] demonstrated the advantage of a multi-model ensemble system over any one model system. In the near term, I would advocate the development of the EUROSIP system to provide information about regional climate change, for climate adaptation on multi-decadal timescales. To deny society the value of such a system because EUROSIP is designed primarily so solve an initial-value problem, and not a forced climate-change problem, would in my view be short-sighted.

On the longer term, we need to look for more radical solutions. The next section discusses one.

## 5. The Curie, Einstein and Lorentz vision for the future of meteorology

Einstein was a childhood hero of mine. His theory of General Relativity combines physical insight and mathematical elegance, unsurpassed in theoretical physics.

And so it was with a great sense of excitement that I read about Einstein’s 1926 letter co-written with Marie Curie and Henrik Lorentz—he of the eponymous Lorentz transformation in special relativity theory—recommending that an ‘International Bureau of Meteorology’ be established [24]. One can hardly think of a more prestigious set of physicists of the time! Einstein himself was a self-acclaimed internationalist. However, in this matter he was almost certainly influenced by Lorentz, a very close colleague, who was President of the League of Nations Commission for International Intellectual Cooperation. In his book ‘The world as I see it’ [25], Einstein commends Lorentz’s contributions in the sphere of international organization and politics, which, Einstein notes, demands a ‘freedom from national prejudice and a devotion to the common ends of all’. Such qualities remain true today.

The publication of this letter did not directly lead to the founding of an international bureau of meteorology. However, 50 years later, such a bureau was established: the ECMWF [26] an international meteorological organization based in Reading UK, and funded by 34 member states. Almost since the first day it produced an operational forecast, ECMWF has been *the* world leading organization for medium-range weather forecasts (out to about two weeks ahead). ECMWF provides a work environment that can attract some of the best scientists from around Europe, has a sense and singularity of purpose and has clear ways to measure success. These ingredients have made ECMWF the envy of meteorological services on other continents and is an outstanding example of what can be achieved if we pool human and computing resources. (Of course, the existence of ECMWF does not in any way reduce the need for national meteorological services, which disseminate ECMWF products nationally, provide and train staff for ECMWF, and add a higher level of detail from regional high-resolution meteorological models run locally, than is possible from the direct global model output.) This success has completely vindicated Curie, Einstein and Lorentz’s vision for such an organization.

And yet we have not achieved such international collaboration in the field of climate prediction. Why is this? Perhaps the most vocal argument is that having an ensemble of quasi-independent nationally developed climate models around the globe is a good thing: it engenders a sense of competition between different institutes and this competitive spirit ensures progress. Moreover, by not ‘putting all our eggs in one basket’ this ensemble of models provides robust estimates of uncertainty in our predictions—surely also a good thing. I do not accept these arguments for a number of reasons.

Of course, if the nations of the world each had infinite resources, there would be nothing wrong with such a strategy. But we do not live in such a world. Indeed, as models get more and more complex—encompassing not only higher and higher resolution, but more and more of the Earth System, and in ensemble rather than deterministic mode—the human and computing resources needed to develop and run these models at the institute level is becoming more and more of a challenge. And these days, a credible climate model is not just a set of modules for the different components of the Earth System, it is a piece of code that attempts to run as efficiently as possible on the many hundreds of thousands of individual processors that comprise a modern supercomputer. Increasingly, climate modellers have to be aware of the hardware that their models run on. And ‘running’ the model is only half the story: writing the output and developing the tools that can efficiently analyse this output is becoming increasingly hard. Because of this, the need for pooling human and computing resources has never been stronger.

I am also not convinced by the argument that having multiple quasi-independent models is necessary to engender competition and hence progress. Having worked for many years at ECMWF, I do not believe that rivalry with other weather forecast centres was the primary reason why the ECMWF scientists strove to produce yet better models. Rather, they wanted to improve on the current ECMWF model. They knew what the shortcomings of the current model were, and they were motivated to produce a new version of the model where these shortcomings were reduced and where the forecast skill scores were improved. This is probably true elsewhere.

I also reject the idea that we necessarily need a large ensemble of quasi-independent models to generate credible estimates of forecast uncertainty. Crucially, I do not see a climate model as a deterministic piece of computer code. Yes, the equations of motion are deterministic, but this does not mean that computational representations of these equations should be deterministic. I have written about these extensively elsewhere (e.g. [27]), and do not intend to spend much space repeating the arguments here. Suffice it to say, that if we close the computational equations with deterministic parametrization formulae, we are doing damage to the scaling symmetries that the underlying partial differential equations respect. It is better to think of these closure equations in terms of random draws from some underlying probability distribution functions, constrained by the grid-scale information. That is to say, a model with inherent stochasticity in its closure schemes is a better representation of the underlying equations than one where the parametrization is a deterministic bulk formula entirely slaved to the grid-scale flow and predicated on some putative—but often non-existent—large ensemble of sub-grid processes.

Stochastic parametrization provides an inbuilt representation of model uncertainty. Is a single model with stochastic parametrization better than a multi-model ensemble? This was studied by Weisheimer *et al.* [28] using monthly and seasonal timescale forecasts. Results overall favoured the single model with stochastic parametrization, though for surface temperature on the seasonal timescale, the multi-model ensemble still had the edge. New stochastic representations of unresolved processes in land surface and oceanic models may swing the pendulum in favour of stochastic parametrization in the coming years.

Stochastic parametrization has become a well-established technique in numerical weather prediction. The reasons are not so much theoretical—a respect of scaling symmetries and so on—but rather that without such stochasticity, ensemble weather forecast systems are generically underdispersive, especially in the tropics where the impact of sub-grid parametrization is large. Stochastic parametrization is not so well established in climate prediction—where researchers prefer to trust estimates of uncertainty produced by (e.g. CMIP) multi-model ensembles. The development of more seamless prediction systems, exploiting synergies between weather and climate prediction, may see stochastic parametrization techniques becoming more established in the climate arena. When this happens, I believe the case for large multi-climate model ensembles will be weakened considerably.

Of course, converging on a single ‘world model’, even if it were stochastic, is unrealistic. Maybe the nations of the world could group together according to their geographical location—thus reducing the number of climate models to roughly one per continent. However, this may be politically naive—some countries get on less well with their neighbours than with distant countries. Perhaps a better analogy is with the groupings by which that the world’s airlines organize themselves. The reason why a particular airline belongs to the One World alliance rather than the Star alliance may be arcane. However, the reasons notwithstanding, we can take advantage of these groupings when trying to make our travel plans as uncomplicated as possible. Similarly, the reasons why individual climate institutes make alliances with others are their own affairs, if such alliances lead to a rationalization of climate modelling. So how many such alliances do we need? I would say as many as possible, given sustained funding for each of around $100 million per year.

Whether all of this needs to be ‘new money’ can be debated, which in turn raises a key question. Research council funding (national and international) for projects is often predicated on having a clear ‘path to impact’. There is a tendency for this to favour projects where partnerships with impact communities are developed within the projects. By contrast, a project to develop a global cloud-resolved climate model may be unable to develop clear partnerships with impact communities for many years—in any case, exascale computing is unlikely to arrive until 2023 or thereabouts and so the fruits of a global cloud-resolved model would not be ready to harvest until then. Indeed, worse than this, insisting prematurely on partnerships with impact communities could be detrimental to such a project, because it would take resources away from the critical goal—the development of one or more climate system models that will be able to serve the impact community (and society more generally) better than any current climate model is able. I would hope that Research Councils will be able to contribute significantly to a strong international programme to develop global cloud-resolved climate models, recognizing that the path to impact would be something that could not happen in the early phases of the project—but in the long run would be almost completely self-apparent.

I have discussed the case for pooling resources for building high-resolution climate models with next-generation exascale computing elsewhere [29]. In this context, one can perhaps make the point that the essential stochasticity of the closure problem for climate, as discussed above, does not require future generation supercomputers to be the paragons of determinism and precision that they have been in the past. This is particularly relevant when one considers that supercomputing performance is now limited by power consumption, not FLOP rate. Essentially, most of this power is needed to move data around in the supercomputer—making a climate model bandwidth limited. As discussed in [30], relaxing the requirements for determinism and precision (where it is not needed) could allow one to increase model resolution without increasing power consumption. However, this will require some changes to current hardware configurations and design. Possible synergies with other areas of computational science (e.g. astrophysics, plasma physics and neuroscience) may help speed up the developments of new types of hybrid computing hardware with variable levels of precision and determinism.

One of the first calls for a more collaborative approach to model development arose from the World Modelling Summit for Climate Prediction [31]. A recent report from the US National Research Councils [32] also calls for a consolidation of climate modelling centres, and better links between weather and climate. With a number of colleagues here in Europe, I am currently writing a framework document for the creation of a new European Flagship Programme on Extreme Computing and Climate, designed to fund a significant new effort to create one or more global cloud-resolved climate models, to run the new generation of exascale computers when they emerge in the next decade, with the specific focus of understanding and simulating climate extremes (for example, understanding the drivers of the persistent jet-stream anomalies that caused extensive flooding over the UK in 2013/2014 and again in 2015/2016). In partnership with the existing climate centres, an initial goal will include the development of one or more global climate models with a global 1 km grid to allow deep convective cloud systems to be partially resolved (and convective parametrizations switched off). However, research with limited domain models suggest that ultimately we should be striving for resolutions of a few hundred metres or less. This may seem unfeasible in the foreseeable future. However, if code can be developed where the bit lengths of model variables can be made to vary with spatial scale (consistent with the dependence of atmospheric Lyapunov exponents on horizontal scale [33]), then such a goal may become viable in the next decade. A formal presentation will be made to national and international funding agencies for such a programme in the coming year or so.

Such a programme will feed directly into the work of the national meteorological and oceanographic services, as they advise government about future climate. The programme will also allow climate scientists who strive for a greater understanding of the drivers of extreme climate, to pursue their science. For example, it will be important that any future cloud-resolved model can be configured in idealized Earth-like configurations (e.g. the so-called aqua-planet). The relationship with academics would be expected to mirror that happening in the high-energy physics community.

## 6. Conclusion

At the 2015 Paris Climate Conference, leaders from 194 countries of the world unanimously acknowledged the serious threat posed by anthropogenic emissions of greenhouse gases; surely, there can no longer be any serious doubt about the reality of this threat. Item 7c of Article 7 of the Paris Climate Agreement (Item 7c of Article 7) recommends: ‘Strengthening scientific knowledge on climate, including research, systematic observation of the climate system and early warning systems, in a manner that informs climate services and supports decision making’. I myself would put it more strongly: Climate science must now step up a gear to provide reliable estimates of the climate of the coming decades, including climate extremes, on both global and regional scales, which are as sharp as possible. But we cannot do this without a step change in our modelling capabilities, and my view is that this will not be achieved without a more collaborative approach to climate model development. However, climate scientists must first come together and decide, independent of the politics of international collaboration and the politics of climate change, whether the scientific case for a more collaborative approach to climate model development can be made. If the climate research community can speak with one voice—as it does, for example, in the IPCC assessment reports—then the job is half done.
